# Bloody Urine

**Published:** 1874-05

**Authors:** 


### BLOODY URINE.

This is always a dangerous symptom, coming on sometimes by very slow degrees. Sometimes the urine appears of a reddish tinge from internal fever or a high state of biliousness, causing some unnecessary alarm. Take a teaspoonful each of oil of turpentine and tincture of guiacum, shake it well, then pour it into the urine. The guiacum falls to the bottom in fine powder, white, yellowish or green. If blood is present this deposite is blue, sometimes as indigo. Some persons keep themselves in a constant state of uneasiness by inspecting urine. This is always unprofitable, for only a physician can have a proper idea of what the quantity or color should be under the circumstances of any given case. In a healthy state of the body, the colder the weather the clearer is the urine, unless a person has exercised a great deal; for then in proportion to the perspiration the more watery particles are carried off, leaving the remainder of a high color, and having more sediment. The more one perspires, the less urine there is. Free urination, other things being equal, is an indication of good health. In several diseases, in cholera, for example, increased urination is a sign of recovery.

